# Alpinumisoflavone

**DOI:** 10.1107/S1600536808006867

**Published:** 2008-03-14

**Authors:** Jerry Joe Ebow Kingsley Harrison, Youhei Tabuchi, Hiroyuki Ishida, Robert Kingsford-Adaboh

**Affiliations:** a Department of Chemistry, Faculty of Science, University of Ghana, Box LG56 Legon, Accra, Ghana; b Department of Chemistry, Faculty of Science, Okayama University, Okayama 700-8530, Japan

## Abstract

The title compound, C_20_H_16_O_5_, {systematic name: 5-hydr­oxy-7-(4-hydroxy­phen­yl)-2,2-dimethyl-2*H*,6*H*-benzo[1,2-*b*:5,4-*b*′]dipyran-6-one}, was obtained by demethyl­ation of the biologically active related compound, 4-*O*-methyl­alpinum­iso­flavone. The mol­ecular structure of the title compound is characterized by a fused tricyclic system that contains an approximately planar benzopyrone ring fragment. The six membered pyran ring adopts a half-chair conformation. Both ring systems show an out-of-plane twist. The dihedral angle between the mean plane of the benzopyrone system and the benzene ring is 54.29 (3)°. The mol­ecules are linked by O—H⋯O hydrogen bonds, forming a mol­ecular tape running along the *b* axis.

## Related literature

For related compounds, see: Kingsford-Adaboh *et al.* (2001 ▶, 2006 ▶). For ring puckering analysis, see: Cremer & Pople (1975 ▶).
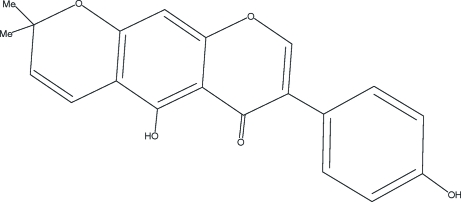


## Experimental

### 

#### Crystal data

C_20_H_16_O_5_
*M* *_r_* = 336.34
Monoclinic, 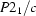

*a* = 13.8333 (3) Å
*b* = 5.92699 (17) Å
*c* = 19.8352 (4) Å
β = 99.6806 (7)°
*V* = 1603.13 (7) Å^3^
*Z* = 4
Mo *K*α radiation
μ = 0.10 mm^−1^
*T* = 93 (2) K
0.53 × 0.45 × 0.43 mm

#### Data collection

Rigaku R-AXIS RAPIDII diffractometer
Absorption correction: multi-scan (**ABSCOR**; Higashi, 1995 ▶) *T*_min_ = 0.771, *T*_max_ = 0.958
30574 measured reflections
4676 independent reflections
4296 reflections with *I* > 2σ(*I*)
*R*_int_ = 0.036

#### Refinement

*R*[*F* ^2^ > 2σ(*F* ^2^)] = 0.042
*wR*(*F* ^2^) = 0.123
*S* = 1.04
4676 reflections
237 parameters
H atoms treated by a mixture of independent and constrained refinement
Δρ_max_ = 0.51 e Å^−3^
Δρ_min_ = −0.26 e Å^−3^

### 

Data collection: *PROCESS-AUTO* (Rigaku/MSC, 2004 ▶); cell refinement: *PROCESS-AUTO* ; data reduction: *CrystalStructure* (Rigaku/MSC, 2004 ▶); program(s) used to solve structure: *SHELXS97* (Sheldrick, 2008 ▶); program(s) used to refine structure: *SHELXL97* (Sheldrick, 2008 ▶); molecular graphics: *ORTEP-3* (Farrugia, 1997 ▶); software used to prepare material for publication: *CrystalStructure*  and *PLATON* (Spek, 2003 ▶).

## Supplementary Material

Crystal structure: contains datablocks global, I. DOI: 10.1107/S1600536808006867/fb2087sup1.cif

Structure factors: contains datablocks I. DOI: 10.1107/S1600536808006867/fb2087Isup2.hkl

Additional supplementary materials:  crystallographic information; 3D view; checkCIF report

## Figures and Tables

**Table 1:** Hydrogen-bond geometry (Å, °)

*D*—H⋯*A*	*D*—H	H⋯*A*	*D*⋯*A*	*D*—H⋯*A*
O2—H2*O*⋯O3	0.92 (2)	1.76 (2)	2.6023 (10)	152.2 (17)
O5—H5*O*⋯O3^i^	0.871 (18)	1.943 (18)	2.7823 (10)	161.4 (17)

Symmetry code: (i) 
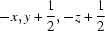
.

**Fig. 1.**
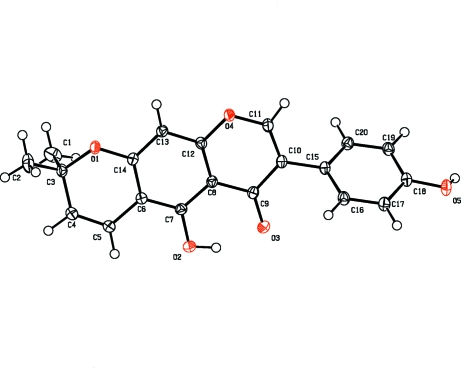
The molecular structure of the title compound. The displacement ellipsoids are drawn at the 50% probability level.

**Fig. 2.**
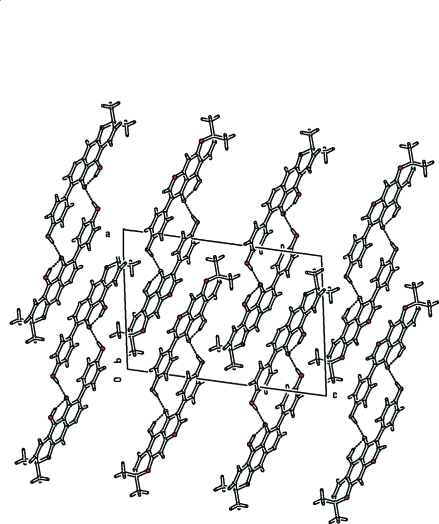
Crystal packing viewed on the a-c plane. Hydrogen bonds are shown as broken lines.

## supplementary crystallographic information

### Comment

The 4-*O*-methylalpinumisoflavone, *O*,*O*-dimethylalpinumisoflavone and 5-*O*-methyl-4-*O*-(3-methylbut-2-en-1-yl)alpinumisoflavone are some of the solvent-extracted compounds from the rootbark and seeds of *Milletia thonningii* whose crystal structures have been studied for obtaining fundamental information on their chemical and biological properties (Kingsford-Adaboh *et al.*, 2001, 2006). These compounds have shown considerable brine shrimp lethality (Kingsford-Adaboh *et al.*, 2006).

In the present work, single crystals of alpinumisoflavone suitable for X-ray diffraction were obtained by demethylation of 4-*O*-methylalpinumisoflavone using cold BCl_3_. The crystals isolated from the crude extract were usually of poor quality. Therefore we decided to modify 4-*O*-methylalpinumisoflavone chemically by demethylation (Scheme 2) hoping that a new compound would yield crystals of a better quality. This turned to be true. The molecular structure of the title compound differs from 4-*O*-methylalpinumisoflavone only in the replacement of the methoxy group by the hydroxyl on the benzene ring D.

The molecular structure of the title compound is characterized by a tricyclic fused ring system, A/B/C, and a benzene ring D (Fig. 1). The benzopyrone ring fragment, B/C, is planar and it is twisted out of plane with respect to the benzene ring D. The outer six-membered ring A is deformed into a half-chair conformation, with Cremer & Pople (1975) parameters q_2_, q_3_ and φ_2_ of 0.2342 (9), -0.1148 (9) Å and 220.4 (2)°, respectively.

The presence of the hydroxyl group proximal to the keto group on the ring C permits the formation of a relatively stronger intramolecular O—H···O hydrogen bond (Table 1). This distance is comparable to the intramolecular contact distance equal to 1.724 (17) Å in 4-*O*-methylalpinumisoflavone which is the closest related compound in the studied series (Kingsford-Adaboh *et al.*, 2001, 2006). The corresponding distances observed in other members of the series are longer (*ca* 2.3 Å; Kingsford-Adaboh *et al.*, 2006).

The observed intramolecular contact is affected by the coplanar arrangement between the hydroxyl and the carbonyl groups in the pertinent part of the molecule. This is demonstrated by the observed torsion angle C9—C8—C7—O2 = -1.19 (13)° in the title structure. The largest deviation from the coplanarity in the series is observed in *O*,*O*-dimethylalpinumisoflavone with the corresponding angle equal to -12.83 (18)° (Kingsford-Adaboh *et al.*, 2001). The intermolecular O—H···O hydrogen bond (Table 1), where the terminal OH group of the benzene ring D serves as a proton donor to the carbonyl oxygen atom, is observed to play an important role in the molecular bonding in the crystal structure (Tab. 1, Fig. 2).

### Experimental

Alpinumisoflavone was obtained from the demethylation of 4-O-methylalpinumisoflavone. Solvent extraction of 4-O-methylalpinumisoflavone from the pulverized root bark of Milletia thonningii followed similar procedure as described in our earlier work (Kingsford-Adaboh et al., 2001). A cold solution of BCl_3_ in chloroform (-78 °C) was added slowly to about 15 ml of a chloroform solution of 4-O-methylalpinumisoflavone (200 mg, 0.571 mmol) cooled to -78 °C using dry ice and acetone mixture. The solution was stirred for about 10 min under argon atmosphere. 30 ml of water was added slowly and the resulting yellowish mixture was extracted with chloroform three times. The combined extracts were washed with water twice and then dried over anhydrous sodium sulphate. After evaporation of the solvent under vacuum, the residue was chromatographed on a silica gel using petroleum ether and ethylacetate mixture in the ratio of between 8/1 and 5/1 as the mobile phase. The product was recrystallized from acetonitrile. The demethylation yield (159.6 mg about 80%, m.p. 486 K). The product was confirmed by ^13^C NMR spectra of both the reactants and the product.)

### Refinement

All the H atoms were located in the difference Fourier map. The H atoms that have been attached to the C atoms were constrained in idealized geometry while the hydroxyl H atoms were freely isotropically refined. C_methyl_—H= 0.98 Å allowing for rotation around the C—C bond with *U*_iso_(H_methyl_) = 1.5*U*_eq_(C_methyl_). C_aryl_—H = 0.95 Å with *U*_iso_(H_aryl_) = 1.2*U*_eq_(C_aryl_).

### Crystal data

**Table:**

C_20_H_16_O_5_	*F*(000) = 704.00
*M**_r_* = 336.34	*D*_x_ = 1.393 Mg m^−^^3^
Monoclinic, *P*2_1_/*c*	Melting point: 486 K
Hall symbol: -P 2ybc	Mo *K*α radiation, λ = 0.71075 Å
*a* = 13.8333 (3) Å	Cell parameters from 27371 reflections
*b* = 5.92699 (17) Å	θ = 3.0–30.0°
*c* = 19.8352 (4) Å	µ = 0.10 mm^−^^1^
β = 99.6806 (7)°	*T* = 93 K
*V* = 1603.13 (7) Å^3^	Block, yellow
*Z* = 4	0.53 × 0.45 × 0.43 mm

### Data collection

**Table:**

Rigaku R-AXIS RAPIDII diffractometer	4296 reflections with *I* > 2σ(*I*)
Detector resolution: 10.00 pixels mm^-1^	*R*_int_ = 0.036
ω scans	θ_max_ = 30.0°, θ_min_ = 3.3°
Absorption correction: multi-scan (*ABSCOR*; Higashi, 1995)	*h* = −19→17
*T*_min_ = 0.771, *T*_max_ = 0.958	*k* = −8→8
30574 measured reflections	*l* = −27→27
4676 independent reflections	

### Refinement

**Table:**

Refinement on *F*^2^	Secondary atom site location: difference Fourier map
Least-squares matrix: full	Hydrogen site location: difference Fourier map
*R*[*F*^2^ > 2σ(*F*^2^)] = 0.042	H atoms treated by a mixture of independent and constrained refinement
*wR*(*F*^2^) = 0.123	*w* = 1/[σ^2^(*F*_o_^2^) + (0.0785*P*)^2^ + 0.3851*P*] where *P* = (*F*_o_^2^ + 2*F*_c_^2^)/3
*S* = 1.04	(Δ/σ)_max_ < 0.001
4676 reflections	Δρ_max_ = 0.51 e Å^−^^3^
237 parameters	Δρ_min_ = −0.26 e Å^−^^3^
0 restraints	Extinction correction: *SHELXL*, Fc^*^=kFc[1+0.001xFc^2^λ^3^/sin(2θ)]^-1/4^
54 constraints	Extinction coefficient: 0.015 (2)
Primary atom site location: structure-invariant direct methods	

### Special details

**Table:**

Geometry. All e.s.d.'s (except the e.s.d. in the dihedral angle between two l.s. planes) are estimated using the full covariance matrix. The cell e.s.d.'s are taken into account individually in the estimation of e.s.d.'s in distances, angles and torsion angles; correlations between e.s.d.'s in cell parameters are only used when they are defined by crystal symmetry. An approximate (isotropic) treatment of cell e.s.d.'s is used for estimating e.s.d.'s involving l.s. planes.
Refinement. Refinement of *F*^2^ against ALL reflections. The weighted *R*-factor *wR* and goodness of fit *S* are based on *F*^2^, conventional *R*-factors *R* are based on *F*, with *F* set to zero for negative *F*^2^. The threshold expression of *F*^2^ > σ(*F*^2^) is used only for calculating *R*-factors(gt) *etc*. and is not relevant to the choice of reflections for refinement. *R*-factors based on *F*^2^ are statistically about twice as large as those based on *F*, and *R*- factors based on ALL data will be even larger.Although there were present diffractions that violated the space-group-systematic absences the average *I*/σ values for *h*0*l* with l = 2n and for l =2n+1 were 25.6 and 0.8, respectively. This indicates presence of the *c* glide plane. Thus we selected *P*2_1_/*c*. We have also refined the structure with *P*2_1_. All atoms except H completely fit to the *c* and *i* symmetries. The reflections that should be absent for *P*2_1_/*c* might be accidentally observed. Probably some of them were diffractions from small ice particles (frost) generated in the X-ray beam path.

### Fractional atomic coordinates and isotropic or equivalent isotropic displacement parameters (Å^2^)

**Table:**

	*x*	*y*	*z*	*U*_iso_*/*U*_eq_	
O1	0.70895 (5)	0.49566 (11)	0.41410 (3)	0.01881 (15)	
O2	0.42098 (5)	0.04434 (11)	0.39657 (4)	0.02165 (16)	
O3	0.26668 (5)	0.18561 (11)	0.31384 (3)	0.02019 (15)	
O4	0.42341 (4)	0.74216 (11)	0.27339 (3)	0.01861 (15)	
O5	−0.12257 (5)	0.49357 (12)	0.12202 (4)	0.02212 (16)	
C1	0.74810 (9)	0.50860 (18)	0.53739 (5)	0.0300 (2)	
H1A	0.7776	0.6578	0.5344	0.045*	
H1B	0.7814	0.4307	0.5784	0.045*	
H1C	0.6784	0.5257	0.5401	0.045*	
C2	0.86499 (7)	0.3562 (2)	0.46408 (6)	0.0312 (2)	
H2A	0.8685	0.2740	0.4217	0.047*	
H2B	0.9035	0.2764	0.5028	0.047*	
H2C	0.8915	0.5086	0.4611	0.047*	
C3	0.75883 (6)	0.37155 (15)	0.47438 (4)	0.01759 (17)	
C4	0.71488 (7)	0.14234 (16)	0.47920 (5)	0.0231 (2)	
H4	0.7539	0.0268	0.5033	0.028*	
C5	0.62256 (7)	0.09525 (16)	0.45064 (5)	0.02201 (19)	
H5	0.5948	−0.0474	0.4580	0.026*	
C6	0.56482 (6)	0.26353 (14)	0.40815 (4)	0.01600 (17)	
C7	0.46669 (6)	0.23138 (14)	0.37950 (4)	0.01593 (17)	
C8	0.41581 (6)	0.38972 (14)	0.33266 (4)	0.01501 (16)	
C9	0.31511 (6)	0.35590 (14)	0.30076 (4)	0.01544 (16)	
C10	0.27298 (6)	0.53058 (15)	0.25305 (4)	0.01599 (16)	
C11	0.32922 (6)	0.71046 (16)	0.24342 (4)	0.01798 (17)	
H11	0.3000	0.8244	0.2130	0.022*	
C12	0.46784 (6)	0.58219 (14)	0.31783 (4)	0.01550 (17)	
C13	0.56476 (6)	0.62234 (15)	0.34640 (4)	0.01663 (17)	
H13	0.5977	0.7550	0.3357	0.020*	
C14	0.61209 (6)	0.46139 (15)	0.39122 (4)	0.01554 (16)	
C15	0.16979 (6)	0.52188 (15)	0.21756 (4)	0.01598 (17)	
C16	0.13347 (6)	0.33720 (15)	0.17706 (4)	0.01859 (18)	
H16	0.1757	0.2145	0.1715	0.022*	
C17	0.03611 (7)	0.33194 (16)	0.14484 (5)	0.01923 (18)	
H17	0.0121	0.2060	0.1174	0.023*	
C18	−0.02636 (6)	0.51134 (15)	0.15274 (4)	0.01714 (17)	
C19	0.00911 (6)	0.69858 (15)	0.19145 (5)	0.01829 (17)	
H19	−0.0328	0.8227	0.1959	0.022*	
C20	0.10686 (6)	0.70239 (15)	0.22366 (4)	0.01783 (17)	
H20	0.1311	0.8300	0.2502	0.021*	
H2O	0.3584 (15)	0.054 (3)	0.3729 (10)	0.057 (5)*	
H5O	−0.1591 (13)	0.580 (3)	0.1428 (9)	0.046 (4)*	

### Atomic displacement parameters (Å^2^)

**Table:**

	*U*^11^	*U*^22^	*U*^33^	*U*^12^	*U*^13^	*U*^23^
O1	0.0133 (3)	0.0231 (3)	0.0186 (3)	−0.0020 (2)	−0.0014 (2)	0.0047 (2)
O2	0.0188 (3)	0.0184 (3)	0.0268 (3)	−0.0039 (2)	0.0009 (3)	0.0060 (2)
O3	0.0156 (3)	0.0197 (3)	0.0251 (3)	−0.0029 (2)	0.0029 (2)	0.0019 (2)
O4	0.0145 (3)	0.0206 (3)	0.0195 (3)	−0.0014 (2)	−0.0006 (2)	0.0063 (2)
O5	0.0133 (3)	0.0288 (4)	0.0226 (3)	0.0034 (2)	−0.0018 (2)	−0.0033 (3)
C1	0.0459 (6)	0.0234 (5)	0.0193 (4)	0.0037 (4)	0.0018 (4)	−0.0028 (3)
C2	0.0159 (4)	0.0492 (7)	0.0277 (5)	0.0032 (4)	0.0010 (4)	0.0116 (4)
C3	0.0160 (4)	0.0206 (4)	0.0149 (3)	0.0002 (3)	−0.0010 (3)	0.0012 (3)
C4	0.0228 (4)	0.0178 (4)	0.0258 (4)	0.0007 (3)	−0.0043 (4)	0.0024 (3)
C5	0.0217 (4)	0.0165 (4)	0.0253 (4)	−0.0008 (3)	−0.0031 (3)	0.0030 (3)
C6	0.0156 (4)	0.0158 (4)	0.0162 (4)	0.0000 (3)	0.0013 (3)	0.0007 (3)
C7	0.0157 (4)	0.0154 (4)	0.0170 (4)	−0.0010 (3)	0.0035 (3)	0.0006 (3)
C8	0.0126 (3)	0.0173 (4)	0.0153 (3)	0.0000 (3)	0.0027 (3)	0.0006 (3)
C9	0.0131 (3)	0.0179 (4)	0.0157 (3)	0.0001 (3)	0.0036 (3)	−0.0012 (3)
C10	0.0129 (3)	0.0194 (4)	0.0156 (3)	0.0011 (3)	0.0022 (3)	−0.0005 (3)
C11	0.0139 (4)	0.0219 (4)	0.0175 (4)	0.0009 (3)	0.0008 (3)	0.0025 (3)
C12	0.0149 (4)	0.0174 (4)	0.0144 (3)	0.0008 (3)	0.0027 (3)	0.0020 (3)
C13	0.0147 (4)	0.0182 (4)	0.0169 (4)	−0.0017 (3)	0.0023 (3)	0.0022 (3)
C14	0.0135 (3)	0.0185 (4)	0.0145 (3)	−0.0008 (3)	0.0020 (3)	−0.0005 (3)
C15	0.0126 (3)	0.0197 (4)	0.0155 (3)	0.0014 (3)	0.0020 (3)	0.0000 (3)
C16	0.0158 (4)	0.0200 (4)	0.0196 (4)	0.0036 (3)	0.0020 (3)	−0.0027 (3)
C17	0.0167 (4)	0.0213 (4)	0.0188 (4)	0.0019 (3)	0.0006 (3)	−0.0037 (3)
C18	0.0135 (4)	0.0219 (4)	0.0157 (3)	0.0018 (3)	0.0015 (3)	0.0007 (3)
C19	0.0154 (4)	0.0193 (4)	0.0203 (4)	0.0035 (3)	0.0033 (3)	−0.0008 (3)
C20	0.0153 (4)	0.0188 (4)	0.0195 (4)	0.0007 (3)	0.0032 (3)	−0.0024 (3)

### Geometric parameters (Å, °)

**Table:**

O1—C14	1.3558 (10)	C6—C7	1.3937 (11)
O1—C3	1.4728 (10)	C6—C14	1.4105 (12)
O2—C7	1.3474 (10)	C7—C8	1.4210 (11)
O2—H2O	0.92 (2)	C8—C12	1.4062 (11)
O3—C9	1.2623 (10)	C8—C9	1.4438 (11)
O4—C11	1.3512 (10)	C9—C10	1.4565 (12)
O4—C12	1.3680 (10)	C10—C11	1.3522 (12)
O5—C18	1.3714 (10)	C10—C15	1.4824 (11)
O5—H5O	0.871 (18)	C11—H11	0.9500
C1—C3	1.5186 (13)	C12—C13	1.3856 (11)
C1—H1A	0.9800	C13—C14	1.3904 (12)
C1—H1B	0.9800	C13—H13	0.9500
C1—H1C	0.9800	C15—C20	1.3973 (11)
C2—C3	1.5190 (13)	C15—C16	1.4001 (12)
C2—H2A	0.9800	C16—C17	1.3907 (11)
C2—H2B	0.9800	C16—H16	0.9500
C2—H2C	0.9800	C17—C18	1.3956 (12)
C3—C4	1.4980 (13)	C17—H17	0.9500
C4—C5	1.3366 (12)	C18—C19	1.3911 (12)
C4—H4	0.9500	C19—C20	1.3952 (11)
C5—C6	1.4549 (12)	C19—H19	0.9500
C5—H5	0.9500	C20—H20	0.9500
			
C14—O1—C3	119.91 (7)	O3—C9—C8	121.81 (8)
C7—O2—H2O	105.4 (12)	O3—C9—C10	122.26 (7)
C11—O4—C12	118.85 (7)	C8—C9—C10	115.93 (7)
C18—O5—H5O	110.0 (12)	C11—C10—C9	118.38 (8)
C3—C1—H1A	109.5	C11—C10—C15	119.43 (8)
C3—C1—H1B	109.5	C9—C10—C15	122.13 (7)
H1A—C1—H1B	109.5	O4—C11—C10	125.64 (8)
C3—C1—H1C	109.5	O4—C11—H11	117.2
H1A—C1—H1C	109.5	C10—C11—H11	117.2
H1B—C1—H1C	109.5	O4—C12—C13	116.33 (7)
C3—C2—H2A	109.5	O4—C12—C8	120.45 (7)
C3—C2—H2B	109.5	C13—C12—C8	123.22 (8)
H2A—C2—H2B	109.5	C12—C13—C14	117.57 (8)
C3—C2—H2C	109.5	C12—C13—H13	121.2
H2A—C2—H2C	109.5	C14—C13—H13	121.2
H2B—C2—H2C	109.5	O1—C14—C13	116.35 (7)
O1—C3—C4	111.41 (7)	O1—C14—C6	121.10 (7)
O1—C3—C1	107.68 (7)	C13—C14—C6	122.37 (8)
C4—C3—C1	109.62 (8)	C20—C15—C16	118.66 (8)
O1—C3—C2	104.61 (7)	C20—C15—C10	119.76 (8)
C4—C3—C2	111.49 (8)	C16—C15—C10	121.57 (7)
C1—C3—C2	111.90 (9)	C17—C16—C15	120.50 (8)
C5—C4—C3	122.11 (8)	C17—C16—H16	119.7
C5—C4—H4	118.9	C15—C16—H16	119.7
C3—C4—H4	118.9	C16—C17—C18	120.10 (8)
C4—C5—C6	119.65 (8)	C16—C17—H17	120.0
C4—C5—H5	120.2	C18—C17—H17	120.0
C6—C5—H5	120.2	O5—C18—C19	122.17 (8)
C7—C6—C14	118.38 (8)	O5—C18—C17	117.68 (8)
C7—C6—C5	123.00 (8)	C19—C18—C17	120.14 (8)
C14—C6—C5	118.46 (8)	C18—C19—C20	119.37 (8)
O2—C7—C6	118.43 (8)	C18—C19—H19	120.3
O2—C7—C8	120.39 (7)	C20—C19—H19	120.3
C6—C7—C8	121.18 (8)	C19—C20—C15	121.19 (8)
C12—C8—C7	117.25 (7)	C19—C20—H20	119.4
C12—C8—C9	120.73 (7)	C15—C20—H20	119.4
C7—C8—C9	122.02 (8)		
			
C14—O1—C3—C4	31.87 (11)	C11—O4—C12—C8	−0.83 (12)
C14—O1—C3—C1	−88.36 (10)	C7—C8—C12—O4	−179.76 (7)
C14—O1—C3—C2	152.45 (8)	C9—C8—C12—O4	0.74 (12)
O1—C3—C4—C5	−24.52 (13)	C7—C8—C12—C13	0.13 (13)
C1—C3—C4—C5	94.56 (11)	C9—C8—C12—C13	−179.37 (8)
C2—C3—C4—C5	−140.97 (10)	O4—C12—C13—C14	−179.35 (7)
C3—C4—C5—C6	5.75 (15)	C8—C12—C13—C14	0.76 (13)
C4—C5—C6—C7	−176.66 (9)	C3—O1—C14—C13	163.91 (7)
C4—C5—C6—C14	7.97 (14)	C3—O1—C14—C6	−20.89 (12)
C14—C6—C7—O2	−178.87 (7)	C12—C13—C14—O1	174.83 (7)
C5—C6—C7—O2	5.75 (13)	C12—C13—C14—C6	−0.31 (13)
C14—C6—C7—C8	1.94 (13)	C7—C6—C14—O1	−175.92 (7)
C5—C6—C7—C8	−173.44 (8)	C5—C6—C14—O1	−0.33 (12)
O2—C7—C8—C12	179.31 (7)	C7—C6—C14—C13	−1.02 (13)
C6—C7—C8—C12	−1.51 (12)	C5—C6—C14—C13	174.57 (8)
O2—C7—C8—C9	−1.19 (13)	C11—C10—C15—C20	−53.12 (12)
C6—C7—C8—C9	177.98 (7)	C9—C10—C15—C20	124.00 (9)
C12—C8—C9—O3	−179.25 (8)	C11—C10—C15—C16	126.14 (9)
C7—C8—C9—O3	1.27 (13)	C9—C10—C15—C16	−56.74 (12)
C12—C8—C9—C10	0.40 (12)	C20—C15—C16—C17	−1.39 (13)
C7—C8—C9—C10	−179.08 (7)	C10—C15—C16—C17	179.35 (8)
O3—C9—C10—C11	178.22 (8)	C15—C16—C17—C18	−0.07 (14)
C8—C9—C10—C11	−1.43 (12)	C16—C17—C18—O5	−177.52 (8)
O3—C9—C10—C15	1.07 (13)	C16—C17—C18—C19	1.62 (14)
C8—C9—C10—C15	−178.58 (7)	O5—C18—C19—C20	177.44 (8)
C12—O4—C11—C10	−0.31 (13)	C17—C18—C19—C20	−1.67 (13)
C9—C10—C11—O4	1.47 (14)	C18—C19—C20—C15	0.18 (13)
C15—C10—C11—O4	178.70 (8)	C16—C15—C20—C19	1.34 (13)
C11—O4—C12—C13	179.27 (7)	C10—C15—C20—C19	−179.39 (8)

### Hydrogen-bond geometry (Å, °)

**Table:**

*D*—H···*A*	*D*—H	H···*A*	*D*···*A*	*D*—H···*A*
O2—H2O···O3	0.92 (2)	1.76 (2)	2.6023 (10)	152.2 (17)
O5—H5O···O3^i^	0.871 (18)	1.943 (18)	2.7823 (10)	161.4 (17)

Symmetry codes: (i) −*x*, *y*+1/2, −*z*+1/2.
